# Secondhand Smoke Exposure and Coronary Artery Calcification among Nonsmoking Participants of a Population-Based Cohort

**DOI:** 10.1289/ehp.1003347

**Published:** 2011-07-08

**Authors:** Frank Peinemann, Susanne Moebus, Nico Dragano, Stefan Möhlenkamp, Nils Lehmann, Hajo Zeeb, Raimund Erbel, Karl-Heinz Jöckel, Barbara Hoffmann, on behalf of the Heinz Nixdorf Recall Study Investigative Group

**Affiliations:** 1 Institute for Medical Informatics, Biometry, and Epidemiology, University Duisburg-Essen, Essen, Germany; 2 Institute for Medical Biometry, Epidemiology, and Informatics, University Medical Center of the Johannes Gutenberg University Mainz, Mainz, Germany; 3 Institute of Medical Sociology, Heinrich Heine University, Düsseldorf, Germany; 4 West German Heart Center, University Hospital, University of Duisburg-Essen, Essen, Germany; 5 Leibniz Research Institute for Environmental Medicine and Medical Faculty, University of Düsseldorf, Düsseldorf, Germany

**Keywords:** cardiovascular atherosclerosis, comparative risk assessment, environmental epidemiology, population health, secondhand smoke

## Abstract

Background: Secondhand smoke (SHS) consists of fine particulate matter, carcinogens, and various toxins that affect large parts of the population. SHS increases the risk for acute cardiovascular events and may contribute to the development of atherosclerosis.

Objectives: We investigated the association of SHS with coronary artery calcification (CAC).

Methods: In this cross-sectional analysis, we used baseline data (2000–2003) from 1,766 never-smokers without clinically manifested coronary heart disease, 45–75 years of age, from the Heinz Nixdorf Recall Study, an ongoing, prospective, population-based cohort study in Germany. Self-reported frequent SHS at home, at work, and in other places was assessed by questionnaire. CAC scores were derived based on electron-beam computed tomography. We conducted multiple linear regression analysis using exposure to SHS as the explanatory variable and ln(CAC+1) as the response variable. We conducted logistic regression to estimate the odds ratio (OR) for presence of any CAC.

Results: Frequent exposure to SHS was reported by 21.5% of participants. After adjustment for age, sex, and socioeconomic status, CAC + 1 was 21.1% [95% confidence interval (CI): –5.5%, 55.2%] higher in exposed than in unexposed participants. After adjusting for other cardiovascular risk factors, the association was attenuated (15.4%; 95% CI: –9.6%, 47.2%). SHS exposure was also associated with a CAC score > 0 (fully adjusted OR = 1.38; 95% CI: 1.03, 1.84).

Conclusions: Self-reported frequent exposure to SHS was associated with subclinical coronary atherosclerosis in our cross-sectional study population. Considering the widespread exposure and the clinical relevance of coronary atherosclerosis, this result, if confirmed, is of public health importance.

Secondhand smoke (SHS) is the combination of smoke given off by the burning end of a tobacco product and the smoke exhaled by the smoker. The Germany National Health Survey 1998 estimated a prevalence of 55% for regular exposure to SHS in nonsmokers 18–79 years of age in Germany (Deutsches Krebsforschungszentrum 2005). Exposure to SHS is a relevant health problem; for example, cardiovascular event rates are 20–50% higher in subjects exposed to SHS than in those not exposed, causing an estimated 40,000–50,000 deaths from coronary heart disease (CHD) in the United States each year (U.S. Department of Health and Human Services 2006). Biological mechanisms proposed to explain adverse effects of SHS on the cardiovascular system include the elicitation of oxidative stress, inflammatory responses, activation of platelets, endothelial dysfunction, and changes of the autonomic balance (Barnoya and Glantz 2005).

Studies focusing on clinical events cannot distinguish between the triggering of acute events and chronic processes leading to the development and progression of atherosclerosis as the underlying pathology for cardiovascular events. It is therefore necessary to investigate measures of subclinical atherosclerosis to assess the potential influence on the underlying disease process. Some studies suggest that SHS contributes to the development and progression of atherosclerosis. Recent studies found associations between SHS and carotid artery intimal-medial thickness (Knoflach et al. 2009), aortic plaque (Kiechl et al. 2002), ankle-brachial index (He et al. 2008), arterial stiffness (Mack et al. 2003), and endothelial function (Leone and Balbarini 2008) as indicators of subclinical atherosclerosis, but not all studies are consistent with a positive association (Agarwal 2009). Whether SHS is associated with coronary atherosclerosis has not yet been investigated. Coronary artery calcification/calcium (CAC) detected by electron-beam computed tomography is a reliable measure of the degree of coronary atherosclerosis and is associated with cardiac events (Erbel et al. 2010; Folsom et al. 2008; Greenland et al. 2007). The aim of the present study was to investigate the association of exposure to SHS and the degree of CAC as a measure of coronary atherosclerosis in participants of a population-based cohort in Germany.

## Materials and Methods

**Figure 1:**
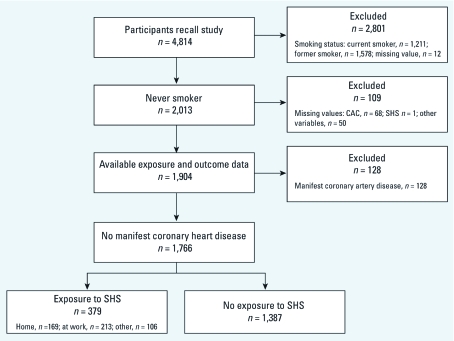
Definition of study population.

*Study population.* We investigated baseline data from an ongoing prospective population-based Caucasian cohort study in Germany (Heinz Nixdorf Recall Study). Details about design (Schmermund et al. 2002) and about baseline recruitment and population-based assessment of subclinical atherosclerosis (Schmermund et al. 2006) have been previously reported. In short, baseline data from 4,814 participants 45–75 years of age were collected from 2000 to 2003. The original cohort aims to clarify the predictive value of coronary calcification for future cardiac events in a random population sample. We used baseline data from 1,766 never-smokers (34% men) with available information on exposure and outcome (Figure 1). Data from 128 participants with clinically manifested CHD (previous myocardial infarction or coronary revascularization) were excluded from the main analysis. The study protocol was approved by the local institutional review board of the University Hospital of Essen, and all participants gave written informed consent before the study.

*Exposure assessment.* Exposure to SHS at the time of the baseline examination was assessed in a computer-aided personal interview: “Do you frequently stay in rooms where people smoke at work, at home, or at other places?” and “How many persons live and smoke in your home including yourself?” We constructed an exposure variable regarding exposure to SHS at any place (yes/no) combining information about the three possible locations of exposure and also evaluated SHS exposure according to location. Objective quantification of exposure time was not provided.

*Outcome assessment.* Coronary atherosclerosis was investigated through an assessment of the degree of CAC by electron-beam computed tomography (EBCT) (Greenland et al. 2007). The person who performed and interpreted the CAC results was blinded to participants’ characteristics, including SHS status. Noncontrast-enhanced EBCT was performed with a C-150 scanner (GE Imatron, South San Francisco, CA, USA) (Schmermund et al. 2006). The scanner was operated in the single-slice mode with an image acquisition time of 100 msec and a section thickness of 3 mm. Prospective electrocardiogram triggering was done at 80% of the R-R interval. Contiguous slices down to the apex of the heart were obtained. The CAC score was determined using the method of Agatston et al. (1990). An area of CAC was defined as at least four contiguous pixels with a computer tomography density ≥ 130 Hounsfield units. The total CAC score was computed comprising all calcified lesions in the epicardial coronary system. Analyses were performed using a Virtuoso workstation (Siemens Medical Solutions, Forchheim, Germany).

*Risk factor assessment.* The baseline assessment included a self-administered questionnaire, a computer-aided personal interview for personal risk factor assessment [family history of cardiovascular disease, history of hypertension and diabetes, smoking history, medications, socioeconomic status (SES)], laboratory tests, anthropometric measurements (weight, height), and a physical examination according to a standard protocol, including the standardized measurement of brachial artery blood pressure. Current medications were coded according to the Anatomical Therapeutic Chemical Classification Index (World Health Organization Collaborating Centre for Drug Statistics Methodology 2005). Diabetes mellitus was defined as a prior physician diagnosis of diabetes, current use of antidiabetic drugs, random blood glucose ≥ 11 mmol/L (≥ 200 mg/dL), or having fasting blood glucose ≥ 7 mmol/L (≥ 126 mg/dL) at the time of baseline examination. We used information from self-reported medical history of such measurements. Information relevant to individual-level SES included formal education [categorized into four categories, with the highest ≥ 18 years (equivalent to a university degree) and the lowest ≤ 10 years (equivalent to a basic school degree and no vocational training)] and employment status (full-time or part-time work of ≥ 15 hr/week, inactive or housewife, pensioner, and unemployed). Leisure time physical activity was assessed by questionnaire and converted into weekly metabolic equivalents, which were included as a continuous variable.

*Statistical analysis.* Analyses were performed on the subgroup of never- smokers (*n* = 1,766) with available information on CAC, SHS exposure, age, sex, length of education, employment status, and other important cardiovascular risk factors [diabetes mellitus, body mass index (BMI), physical activity, and ratio of low- to high-density lipoprotein (LDL:HDL)]. We conducted unadjusted and multiple linear regression analysis with exposure to SHS at any place (yes, no) as the explanatory variable. For these analyses, CAC scores were transformed to normalize the distribution by using ln(CAC + 1) as the response variable. The relative effect of SHS exposure is given as percent change in CAC + 1 associated with any exposure versus none. We used a directed acyclic graph, also known as causal diagram, to identify confounders and factors that may represent intermediate steps in the causal path (Glymour and Greenland 2008; Greenland et al. 1999; Shrier and Platt 2008). We concluded that age, sex, years of education, and employment were necessary covariates, which we used for adjustment in the main model. We also adjusted for other factors, including potential intermediates in extended models, such as cardiovascular risk factors diabetes mellitus, BMI, physical activity, and LDL:HDL, as important predictors of CAC. Because the simultaneous adjustment for SES variables and cardiovascular risk factors that are also related to SES can lead to an overadjustment, we conducted this analysis as an additional step.

Because SHS exposure might exert its effect on subclinical atherosclerosis through an increase in blood pressure, we included systolic blood pressure and antihypertensive medication in a separate step.

To identify the most important location of SHS exposure, we included exposure to SHS at home, at work, and at other places as separate indicator variables in one model. This analysis was repeated after limiting the study population to participants < 66 years of age. In addition, we created indicator variables representing increasing exposure to SHS (no exposure as reference, exposure only at home, and exposure at home plus exposure at work or other places). To visualize the age- and sex-specific difference between exposed and nonexposed subjects, we stratified the regression analysis by exposure status (any SHS or no SHS) and plotted predicted CAC over age in both exposure groups. Separate graphs were generated for men and women, analogous to our prior analysis of the effect of smoking cessation on CAC (Jöckel et al. 2009).

To estimate the effect of SHS on the prevalence of any calcification (CAC > 0), we used logistic regression to derive odds ratios (ORs) for CAC > 0. In addition, we investigated the effect of using different CAC score cut points for defining the dichotomous outcome variable, from CAC > 0 versus CAC = 0, CAC > 1 versus CAC ≤ 1, and so forth, up to CAC > 1,000 versus CAC ≤ 1,000, and repeated the logistic regression analysis with these different response variables. As a further sensitivity analysis, we used ln(CAC + 0.1) and ln(CAC + 10) as the response variable in addition to ln(CAC + 1) in linear regression models. We also estimated the percent change in CAC associated with SHS in an analysis restricted to those with measurable CAC (*n* = 1,150) and estimated associations between SHS and prevalent diagnosed CHD at baseline (128 cases among 1,894 participants). All statistical analyses were conducted using SAS software (version 9.2; SAS Institute Inc., Cary, NC, USA).

## Results

Self-reported frequent exposure to any SHS was reported by 21.5% of 1,766 never- smokers (Figure 1) and varied considerably by age and sex, as shown by a lower median age and higher percentage of men in the exposed group (Table 1). CAC was also strongly associated with age and sex (Table 2). In the age-stratified analyses, SHS-exposed participants generally had higher CAC values and frequencies of CAC > 0 than did nonexposed participants. In the full cohort of never- smokers (*n* = 1,894), which included 128 participants with manifested CHD, CHD was more prevalent among participants reporting SHS exposure (33 of 412, 8.0%) than in those unexposed (95 of 1,482, 6.4%).

**Table 1:** Baseline characteristics of analyzed never-smokers (*n* = 1,766) in the Heinz Nixdorf Recall Study according to SHS exposure status.

Baseline characteristic	SHS*a* (*n* = 379)	No SHS (*n* = 1,387)	Total (*n* = 1,766)
CAC*b*						
Mean		120.9		171.5		160.06
50th percentile (median)		7.0		7.7		7.7
75th percentile		56.2		99.0		88.7
Maximum value		4,790.3		5,636.3		5,636.3
Values > 0 [*n* (%)]		248 (66.4)		902 (65.0)		1,150 (65.1)
Age [years (median)]		57.0		63.0		62.0
BMI [kg/m^2^ (median)]		27.6		27.2		27.3
Physical activity, metabolic equivalents per week (median)		579.2		593.6		591.6
LDL [mg/dL (median)]		146.0		144.0		145.0
HDL [mg/dL (median)]		55.0		57.0		56.0
Systolic blood pressure [mmHg (median)]		131.5		131.5		131.5
Men [*n* (%)]		146 (38.5)		454 (32.7)		600 (34.0)
Diabetes mellitus [*n* (%)]		46 (12.1)		164 (11.8)		210 (11.9)
Antihypertensive drugs [*n* (%)]*c*		123 (32.5)		502 (36.2)		625 (35.4)
Length of education [*n* (%)]						
≤ 10 years		62 (16.4)		212 (15.3)		274 (15.5)
11–13 years		216 (57.0)		761 (54.9)		977 (55.3)
14–17 years		70 (18.5)		260 (18.7)		330 (18.7)
≥ 18 years		31 (8.2)		154 (11.1)		195 (11.0)
Employment status [*n* (%)]						
Full-time or part-time work (≥ 15 hr/week)		194 (51.2)		360 (26.0)		554 (31.4)
Inactive or housewife		54 (14.2)		318 (22.9)		372 (21.1)
Pensioner		103 (27.2)		639 (46.1)		742 (42.0)
Unemployed		28 (7.4)		70 (5.0)		98 (5.5)
No. of smokers at home [*n* (%)]*d*						
0		203 (53.6)		1,284 (92.6)		1,487 (84.2)
1		151 (39.8)		96 (6.9)		247 (14.0)
≥ 2		25 (6.6)		7 (0.5)		32 (1.8)
Location of exposure						
Home		1,597		169		1,766
Work		1,553		213		1,766
Other		1,660		106		1,766
**a**Self-reported frequent exposure to SHS at work, at home, or at other places. **b**Detected by EBCT; mean CAC refers to the arithmetic mean in this population. **c**Data missing for 116. **d**A total of 128 participants lived with a current smoker and reported no SHS at home; 18 participants reported SHS at home and did not live with a current smoker.						

**Table 2:** Distribution of CAC among never-smokers without clinically manifested CHD (*n* = 1,766) according to age group and sex.

SHS*a* (*n* = 379)							No SHS (*n* = 1,387)							Total (*n* = 1,766)						
Category	*n* (%)	P50	P75	CAC > 0 [*n* (%)]	*n* (%)	P50	P75	CAC > 0 [*n* (%)]	*n* (%)	P50	P75	CAC > 0 [*n* (%)]
Age group (years)																								
45 to < 55		143 (37.7)		1.0		17.5		73 (51.0)		286 (20.6)		0.0		7.2		118 (41.3)		429 (24.3)		0.0		12.4		191 (44.5)
55 to < 65		152 (40.1)		9.3		55.5		106 (69.7)		547 (39.4)		6.2		72.7		357 (65.3)		699 (39.6)		7.2		66.3		463 (66.2)
≥ 65		84 (22.2)		42.5		225.9		69 (82.1)		554 (39.9)		42.3		203.5		427 (77.1)		638 (36.1)		42.4		204.7		496 (77.7)
Sex																								
Men		146 (38.5)		36.2		157.6		125 (85.6)		454 (32.7)		49.7		247.8		362 (79.7)		600 (34.0)		47.7		205.9		487 (81.2)
Women		233 (61.5)		1.0		23.5		123 (52.8)		933 (67.3)		2.6		51.8		540 (57.9)		1,166 (66.0)		2.1		44.4		663 (56.9)
Abbreviations: P50, 50th percentile (median); P75, 75th percentile. **a**Self-reported frequent exposure to SHS either at work, at home, or at other places.																								

In linear regression analyses, the unadjusted crude estimate [–18.6%; 95% confidence interval (CI): –38.0%, 7.0%] indicated an inverse association of SHS with CAC + 1 (Table 3). After adjusting for age, sex, years of education, and economic activity, CAC + 1 was 21.1% (95% CI: –5.5%, 55.2%) higher in exposed than in unexposed participants. Further adjustment for the cardiovascular risk factors diabetes mellitus, BMI, physical activity, and LDL:HDL attenuated the association (15.4%; 95% CI: –9.6%, 47.2%). Additional adjustment for potential intermediate variables hypertension and antihypertensive therapy decreased the estimate to 10.6% (95% CI: –14.3%, 42.7%). When limiting the analysis to subjects with CAC > 0 (*n* = 1,150), we did not see an association with SHS (data not shown).

**Table 3:** Results of linear and logistic regression analysis.*a*

Linear regression model		Logistic regression	
Category	Percent change in CAC+1*b *(95% CI)		OR*c *(95% CI)	
Unadjusted model for exposure to any SHS		–18.6	(–38.0%, 7.0%)		1.02	(0.80, 1.29)
Model 1 (age, sex)		22.3	(–4.1%, 56.1%)		1.46	(1.12, 1.92)
Model 2 (age, sex, SES)		21.1	(–5.5%, 55.2%)		1.47	(1.11, 1.94)
45 to < 55 years of age		28.3	(–10.6%, 84.1%)		1.59	(1.01, 2.49)
55 to < 65 years of age		8.1	(–26.6%, 59.2%)		1.37	(0.89, 2.12)
≥ 65 years of age		21.4	(–29.4%, 108.6%)		1.41	(0.75, 2.64)
Men		27.8	(–16.5%, 95.7%)		1.96	(1.11, 3.44)
Women		16.0	(–14.6%, 57.6%)		1.33	(0.96, 1.85)
Model 3 (age, sex, SES, diabetes mellitus, BMI, physical activity, LDL:HDL)		15.4	(–9.6%, 47.2%)		1.38	(1.03, 1.84)
Model 4*d* (age, sex, SES)		21.1	(–6.5%, 56.8%)		1.41	(1.06, 1.89)
Model 5*d* (age, sex, SES, hypertension and antihypertensive therapy)		10.6	(–14.3%, 42.7%)		1.30	(0.97, 1.74)
SES comprises two covariates: years of education and employment status. **a**Analysis included 1,766 never-smokers without clinically manifested CHD for models 1–3 and 1,650 never-smokers for models 4 and 5. Manifested CHD includes previous myocardial infarction or coronary revascularization. **b**The regression of ln(CAC + 1) can be converted to the percent change in CAC + 1 using the equation [(exp^β^ – 1) × 100], where β is the change in ln(CAC + 1) associated with SHS exposure. **c**Response variable of the logistic regression model: CAC > 0 (*n* = 1,150) versus CAC = 0 (*n* = 616). **d**Smaller sample size (*n* = 1,650) because of missing values for antihypertensive therapy; response variable of the logistic regression model: CAC > 0 (*n* = 1,084) versus CAC = 0 (*n* = 566).						

In logistic regression analysis, the OR for CAC > 0 was 1.47 (95% CI: 1.11, 1.94), adjusted for sex, age, years of education, and employment status, and 1.38 (95% CI: 1.03, 1.84) after also adjusting for diabetes, BMI, physical activity and LDL:HDL (Table 3). Adding the potential intermediate variables hypertension and antihypertensive therapy to the model decreased the OR to 1.30 (95% CI: 0.97, 1.74). Using different cut-points for dichotomizing CAC in a secondary analysis revealed that CAC > 0 versus CAC = 0 up to CAC > 10 versus CAC ≤ 10 had ORs with lower 95% confidence limits > 1.0 (data not shown). Cut-points between CAC > 20 versus CAC ≤ 20 and CAC > 800 versus CAC ≤ 800 resulted in positive effect estimates but with 95% CIs including 1.0.

A linear regression model that included indicator variables for all three possible exposure locations plus age, sex, education, and employment status showed a stronger association between increased CAC + 1 and SHS exposure at home (34.1%; 95% CI: –5.0%, 89.3%) than exposure at work (7.1%; 95% CI: –22.8%, 48.7%) or at other places (–7.6%; 95% CI: –39.4%, 40.9%). We found no clear exposure–response relationship in the adjusted linear regression analysis. Compared with the reference group (no exposure), exposure to SHS only at home and exposure to SHS at home plus exposure at work led to increases in CAC + 1 of 52.1% (95% CI: –3.0%, 118.1%) and 17.9% (95% CI: –28.1%, 93.4%), respectively. Restricting the analysis to participants < 66 years of age did not substantially influence results (data not shown). We chose this cutoff because legal entitlement to a pension is 65 years in Germany. In logistic regression, SHS at home, at work, and at other places was associated with presence of CAC with ORs of 1.21 (95% CI: 0.82, 1.78), 1.24 (95% CI: 0.87, 1.76), and 1.53 (95% CI: 0.93, 2.49), respectively.

We found no clear differences in associations according to age or sex, but point estimates were generally stronger among men than among women (Table 3).

**Figure 2:**
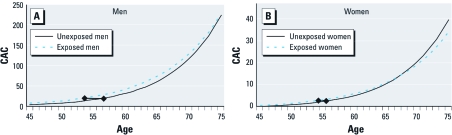
Predicted CAC from multivariable regression of ln(CAC+1) on age and sex: men (*A*) and women (*B*). The diamonds represent the estimated difference in vascular age between SHS-exposed participants and unexposed participants.

Figure 2, A and B, illustrates predicted CAC scores in men and women, respectively, according to age and exposure status. The predicted CAC score for a 45- to 54-year-old man with SHS exposure is comparable to the predicted CAC score for an unexposed man who is about 3 years older, whereas the predicted CAC score for an exposed man 65–74 years of age is comparable to the predicted score for an unexposed man who is about 1 year older. Women had much lower CAC scores than men at all ages, and we observed no clear association between SHS and predicted CAC score at any age.

In sensitivity analyses, the association between SHS and CAC score remained positive when we used ln(CAC + 0.1) and ln(CAC + 10) as dependent variables in linear regression. The age- and sex-adjusted OR for the association of SHS with clinically manifested CHD was 1.48 (95% CI: 0.96, 2.26) and 1.43 (95% CI: 0.92, 2.22), respectively, with further adjustment for diabetes, BMI, physical activity, and LDL:HDL.

## Discussion

This is the first study to examine exposure to SHS and CAC, a measure of subclinical atherosclerosis that is highly predictive of future cardiovascular events (Erbel et al. 2010; Folsom et al. 2008; van der Meer et al. 2004). We used CAC = 0 versus CAC > 0 as dichotomous classification of coronary atherosclerosis in accordance with other studies (Detrano et al. 2008; Greenland et al. 2004). We found that exposure to SHS is associated with prevalence of CAC > 0 in never- smokers. This association is robust to the inclusion of a variety of cardiovascular risk factors. Our results suggest that SHS may increase the risk of cardiovascular disease in part by influencing the underlying chronic process of coronary atherosclerosis, as has been suggested for ambient air pollution (Brook et al. 2010; U.S. Department of Health and Human Services 2006).

The observed association between SHS and CAC is primarily based on the contrast between no calcification and any detectable calcification. Different biological mechanisms could be responsible for the development of plaque, which encompasses earlier stages of atherosclerotic disease, than those leading to the progression of already existing calcified plaque in advanced atherosclerotic disease. Animal experiments support the role of SHS in early stages of atherosclerosis, showing a causal role of SHS in the development of atherosclerotic lesions (Knight-Lozano et al. 2002). Exposure to SHS is associated with permanent inflammation and lipid accumulation in blood vessels in mice (Yuan et al. 2007) and *in utero* exposure results in an increased and earlier adult atherosclerotic lesion formation (Yang et al. 2007). Human studies have shown that the pathophysiology of SHS-induced cardiovascular toxicity includes arterial endothelial dysfunction, platelet activation, oxidation of low-density lipoprotein cholesterol, and increased insulin resistance, which are risk factors for atherosclerosis at early stages of the disease (Barnoya and Glantz 2005). The association between exposure to SHS and atherosclerosis might be initiated as early as during gestation (Geerts et al. 2008). Increased thickening of the coronary walls has been observed in autopsies of fetuses of smoking mothers and of infants of smoking parents (Matturri et al. 2005).

Our inability to observe a linear association of SHS with CAC in the subgroup of participants with more advanced coronary atherosclerosis, as manifested by presence of any CAC, might also be the result of an unfavorable signal-to-noise ratio, with the contribution of SHS to the progression of CAC being overshadowed by the increasing prevalence of other risk factors.

Atherosclerosis and hypertension are distinct but associated disease entities. The attenuation of associations between CAC and SHS after adjustment for blood pressure and antihypertensive medication is consistent with our assumption that high blood pressure may be an intermediate step in the causal pathway. Endothelial dysfunction, reduction of vascular reactivity, and autonomic imbalance might be underlying physiologic pathways leading to an increase in blood pressure, although the exact mechanisms remain to be elucidated. A further potential mechanistic pathway is the elicitation of oxidative stress and the induction of a systemic inflammatory response, which are both related to atherogenesis and cardiovascular events (Libby et al. 2009).

The difference between the crude and adjusted estimates from the linear regression analysis is remarkable and can be explained by confounding by age, which is strongly associated with both exposure and outcome. We restricted covariates in the main model to variables that we defined *a priori* as necessary confounders based on a directed acyclic graph (sex, age, length of education, employment status). Adjusting for other cardiovascular risk factors attenuated the estimated percent change but did not markedly influence the OR for presence of CAC. Adjusting for SES may have reduced confounding due to factors such as antioxidant intake, which is related to SES.

Prior studies have examined the association of SHS with different morphologic markers of atherosclerosis, making direct comparisons across studies difficult. The size of the estimated effect in our study was comparable to a 3-year increase in vascular age in middle-age men, which represents a clinically relevant effect of SHS. In women and in the elderly, the effect on coronary age was diminished. As expected, the estimated effect was much smaller than the effect of active smoking (Jöckel et al. 2009).

One important limitation of this study is its small sample size, leading to an imprecise estimation of effects, manifesting as broad CIs. Even though the complete cohort consists of almost 5,000 participants, we included only never- smokers in the analysis of SHS exposure. Because the prevalence of smoking in Germany is still high, only 39% of the Heinz Nixdorf Recall Study participants were never- smokers. Among these never- smokers, only a relatively low proportion (22%) of participants were considered exposed to SHS, in contrast to an estimated 55% exposure in the general adult population (Deutsches Krebsforschungszentrum 2005). This apparent discrepancy can be explained by three factors: *a*) The type of SHS assessment required frequent exposure to SHS. *b*) Never- smokers are a select population, socializing less with smokers compared with the general population. *c*) We restricted our study population to the age range of 45–74 years, which does not include younger subjects with probable higher and more frequent SHS exposure.

The other major limitation of this study is the comparatively crude assessment of SHS exposure. The question whether participants “frequently stay in rooms where people smoke at work, at home, or at other places” may not clearly distinguish between an exposure that happened only recently and a life-long exposure. Moreover, current exposure does not necessarily reflect long-term past exposure, and the validity of self-reported SHS exposure in general may be compromised by poor recall. The lack of information on the exposure period and the average intensity of exposure to SHS prevented a more refined analysis of the exposure–response relationship and may have contributed to misclassification of exposure (Thun et al. 1999). However, nondifferential misclassification of a binary exposure biases the effect toward the null. Therefore, the true size of the association might actually be higher than estimated in the present analyses. Although we did not take the specific type of occupation into consideration, some of which might be associated with dust exposure and SHS, we adjusted for educational attainment.

The lack of biomarker information such as urinary cotinine is not considered a major limitation in this analysis, because coronary atherosclerosis takes many years to develop and is not influenced by short-term exposure during the days before outcome assessment. However, biomarker information could have helped to validate self-reported smoking status and might have prevented the unintended inclusion of active smokers in the study population.

This study is a cross-sectional analysis and therefore does not allow direct conclusions regarding the development and progression of atherosclerosis. Future analyses in this cohort study with repeated measurements of CAC will investigate the progression of CAC in relation to SHS exposure.

Strengths of the study include the precise and reliable assessment of coronary atherosclerosis (CAC measured by EBCT) and the detailed and standardized assessment of cardiovascular risk factors in a large population-based cohort.

## Conclusions

Self-reported frequent exposure to SHS was associated with subclinical coronary atherosclerosis in our cross-sectional study population. Considering the widespread exposure and the clinical relevance of coronary atherosclerosis, this result, if confirmed, is of public health importance.
